# Effects of storage conditions on oxidative stress biomarkers: methodological implications for ecological and evolutionary studies

**DOI:** 10.1242/jeb.251748

**Published:** 2026-03-05

**Authors:** Francisco Miranda, Francisco Garcia-Gonzalez, Marko Prokić, Miguel Lozano, Antonio Carrillo-Vico, Eduardo Ponce-España, Cristina Álvarez, Francisco J. Arispón, Ivan Gomez-Mestre, Pablo Burraco

**Affiliations:** ^1^ Doñana Biological Station, Avenida Américo Vespucio 26, 41092 Sevilla, Spain; ^2^ Centre for Evolutionary Biology, School of Biological Sciences, University of Western Australia, Crawley 6009, WA, Australia; ^3^ Department of Physiology, Institute for Biological Research “Siniša Stanković” – National Institute of Republic of Serbia, University of Belgrade, Bulevar despota Stefana 142, 11108 Belgrade, Serbia; ^4^ Instituto de Biomedicina de Sevilla, IBiS/Hospital Universitario Virgen del Rocío/CSIC/Universidad de Sevilla, 41013 Seville, Spain; ^5^ Departamento de Bioquímica Médica y Biología Molecular e Inmunología, Facultad de Medicina, Universidad de Sevilla, 41009 Seville, Spain; ^6^ Departamento de Seguridad Alimentaria, PROCAVI S.L. Ctra. Comarcal 339, km 23,6. 41620, Marchena, Seville, Spain

**Keywords:** Biomarker stability, Comparative physiology, Long-term preservation, Redox status, Sample storage, Tissue sensitivity

## Abstract

Understanding oxidative stress in ecological and evolutionary contexts requires reliable biomarker quantification across taxa, tissues and experimental setups. However, storage conditions such as temperature and duration may bias these measurements. Here, we evaluated the stability of oxidative stress biomarkers, including three antioxidant enzymes (superoxide dismutase, glutathione reductase, glutathione peroxidase) and a lipid peroxidation marker (malondialdehyde) in amphibian, mammal, bird and insect samples stored under various temperature conditions (−80, −20, 4°C) from a few hours to 8 months. Storage significantly affected biomarker values depending on the marker, tissue and taxon. Notably, even long-term storage at −80°C altered some markers. In insect samples, lipid peroxidation was also influenced by triglyceride levels, indicating a potential confounding factor. Our results highlight the need to consider storage effects in oxidative stress studies. We also provide practical recommendations, aiming to improve data reliability across field and laboratory eco-evolutionary studies, as well as biomedical contexts.

## Teaser

**Summary:** Storage temperature and duration influence oxidative stress biomarkers across tissues and taxa, identifying potential biases while providing practical guidance for field and laboratory eco-evolutionary studies.

## INTRODUCTION

Understanding the proximate mechanisms underlying life-history strategies is a major challenge in ecology and evolution (Crespi et al., 2013; Flatt and Heyland, 2011). This has become particularly important in the current context of global environmental change, as such knowledge can help disentangle how organisms respond to different scenarios, as well as predict population dynamics and develop effective conservation and restoration strategies (Johnston et al., 2019; Kearney and Porter, 2009; Somero, 2012). Among key mechanisms, oxidative stress has emerged as a central physiological mediator linking environmental conditions with life-history trade-offs (Dowling and Simmons, 2009; Hood et al., 2018; Martin et al., 2024; Metcalfe and Alonso-Alvarez, 2010; Monaghan et al., 2009). As a result, considerable research effort has been devoted to measuring oxidative stress, and the number of studies using oxidative stress as biomarkers parameters has increased exponentially over the last two decades (Costantini, 2014, 2024; Salmón and Burraco, 2022; Speakman et al., 2015). However, methodological limitations, particularly those related to sample storage conditions, can affect the reliability of oxidative stress quantification. Assessing how these conditions affect biomarker stability is therefore essential to ensure robust and comparable results.

Aerobic respiration is the primary process by which organisms generate energy through mitochondrial phosphorylation (Koch et al., 2021). While essential for life, this process leads to the production of reactive oxygen species (ROS) as a by-product of electron leakage in the mitochondrial respiration chain (Halliwell, 2007; Koch et al., 2021; Sies et al., 2017). Although ROS can play functional roles in cell signalling and immune defence, their accumulation beyond a certain threshold can damage different cellular components such as lipids, proteins and DNA (Halliwell, 2007; Sies et al., 2017). To counteract these effects, organisms can activate antioxidant defences, both enzymatic and non-enzymatic; the redox status is the result of ROS production and the action of antioxidant defences (Halliwell, 2007; Sies et al., 2017). When this balance is disrupted, cells experience a state of oxidative stress, which plays a major role in mediating life-history trade-offs and can impair functions closely linked to fitness, including health, reproduction and survival (Metcalfe and Alonso-Alvarez, 2010; Monaghan et al., 2009; Speakman et al., 2015). A causal role of oxidative stress has recently been demonstrated through experiments manipulating oxidative stress pathways, such as in lizards facing heat-wave events or damselflies inducing compensatory growth responses after starvation (Janssens and Stoks, 2020; Zhang et al., 2023). Likewise, the redox machinery plays an important role in explaining the evolution of developmental plastic responses to environmental shifts (Boonekamp et al., 2018; Burraco et al., 2022; Costantini, 2014). Furthermore, the free-radical damage theory of ageing presents oxidative stress as an important driver of ageing and lifespan variation across the tree of life (Gladyshev, 2014; Speakman and Selman, 2011). Oxidative stress is, therefore, deeply involved in many eco-evolutionary processes, which explains the extensive research conducted on oxidative stress pathways across diverse biological contexts.

Markers of oxidative stress are now widely quantified across taxa and systems, in both laboratory and field settings (Costantini, 2024; Costantini et al., 2010). Some of these approaches include longitudinal tracking (i.e. within individuals over time) of oxidative stress markers, which is particularly powerful for investigating life-history trade-offs under different conditions (Selman et al., 2012; Speakman et al., 2015). Likewise, cross-sectional studies provide valuable insights in ecological and evolutionary research, often showing oxidative stress responses to contrasting environments, and measured in tissues that cannot be sampled more than once over the course of a lifetime. Yet, the reliability of such studies depends on the accuracy of the measurements, which can be influenced by methodological factors such as storage temperature and duration (Hõrak and Cohen, 2010). This can be particularly critical for field-based studies, where access to ultra-low temperature freezers is often limited, and thus samples may need to be stored under suboptimal conditions for varying lengths of time (Hõrak and Cohen, 2010). Likewise, sample conservation is crucial in biomedicine (Collard et al., 2013) and the food industry (Tatar, 2024). Identifying how storage conditions affect oxidative stress markers is, therefore, essential to ensure data quality and reliable conclusions within and across studies.

Evidence on how sample storage conditions affect oxidative stress parameters is relatively scarce and fragmented, often focused on specific taxa (mostly humans), tissues or biomarkers, rather than providing a comprehensive framework (Aguilar Diaz De Leon and Borges, 2020; Halliwell and Whiteman, 2004; Jansen et al., 2017; Marrocco et al., 2017). Knowledge is limited for non-human species, with most studies assessing the stability of lipid peroxidation and total antioxidant capacity under different storage conditions (Hõrak and Cohen, 2010; Melvin et al., 2022). Surprisingly, little is known about how post-sampling conditions affect antioxidant enzyme activity, although some studies have focused on gametes within the context of the reproductive health industry (Nair et al., 2006; Peña, 2019).

Here, we aimed to improve our understanding of the influence of temperature and storage duration on the stability of oxidative stress biomarkers across multiple taxa and tissues, and to evaluate how this can potentially influence the outcome of eco-evolutionary studies. Specifically, we measured the activity of three antioxidant enzymes [superoxide dismutase (SOD), glutathione reductase (GR) and glutathione peroxidase (GPX)] and the concentration of malondialdehyde (MDA), which is the most used marker of lipid oxidative damage. We measured these parameters in liver and muscle tissues of an amphibian, a mammal and a bird, and in whole-body samples of an insect. Tissue samples from the same individuals were stored at three different temperatures (−80, −20 and 4°C) for varying durations, ranging from a few hours to up to 8 months. Through this experimental design, we aimed to determine how optimal and suboptimal conditions may influence longitudinal and cross-sectional studies. Additionally, we quantified triglyceride levels to investigate whether the amount of lipids influences estimates of lipid peroxidation (Pérez-Rodríguez et al., 2015; Romero-Haro et al., 2015). As temperature can affect the stability of oxidative stress biomarkers, often leading to increased lipid peroxidation and reduced antioxidant activity during storage (Emekli-Alturfan et al., 2009; Jansen et al., 2017; Rubio et al., 2018; Tsikas, 2017), we expected antioxidant enzyme activity to decrease and lipid peroxide levels to increase at suboptimal temperatures (i.e. 4 and −20°C) compared with −80°C. We also expected temporal changes in marker stability within the same storage temperature, including within −80°C treatments. Because tissues and species differ in biochemical composition, metabolic activity and baseline redox state (Dawson et al., 2025; Halliwell, 2007; Pamplona and Costantini, 2011), we anticipated that the magnitude and direction of storage effects would vary across species and between tissues. Finally, we expected a positive relationship between lipid peroxidation and triglycerides, which may be stronger in the liver as a result of its greater capacity for lipid accumulation compared with muscle. Overall, our study aims to provide methodological insights for ecological and evolutionary research using oxidative stress biomarkers, highlighting how tissue type, species identity and experimental design may influence biomarker reliability.

## MATERIALS AND METHODS

### Animal husbandry and sample collection

Four animal species representing distinct taxonomic groups were used to evaluate how storage conditions affect the stability of oxidative stress biomarkers, as well as the relationship between lipid peroxidation and triglyceride levels. Given the biological and ecological differences among species, appropriate animal husbandry and sample collection procedures were performed. Detailed information for each species is provided below.

We used the spadefoot toad, *Pelobates cultripes* (Cuvier 1829), as a representative amphibian species. In our study, we collected portions of five clutches in southern Spain, and reared the resulting tadpoles under standard conditions for this species, i.e. at 20°C, in 3 l plastic containers filled with dechlorinated water, on a 12 h:12 h light:dark photoperiod and fed *ad libitum* with rabbit chow and spinach (Hyeun-Ji et al., 2020), in climate-controlled chambers at the Doñana Biological Station (Spanish National Research Council, Seville, Spain). After completing metamorphosis, juveniles were kept individually in 3 l buckets filled with a 10 cm layer of humid vermiculite as substrate. Vitamin-dusted crickets were provided *ad libitum* as a food source (Burraco et al., 2023a,b; Kulkarni et al., 2017), and environmental conditions were maintained at 20°C, 40% relative humidity, on a 12 h:12 h light:dark photoperiod. When juveniles reached one year of age, groups of five individuals were placed in 100×50×50 cm terraria containing moist sand, and housed in a climatic chamber set at 20°C, 40% relative humidity, with a 12 h:12 h light:dark photoperiod. At 6 years of age, we randomly selected 10 male individuals from different terraria, anaesthetised them by immersion in MS-222 and then euthanised them via injection of 200 mg ml^−1^ pentobarbital sodium under veterinarian supervision. Finally, we dissected the liver and muscle (from the left hindlimb) of each individual, divided the collected tissues into separate pieces and assigned them to the different storage treatments (see below). All procedures involving amphibians were carried out under ethical approval 21/02/2024/030, granted by the Dirección General de la Producción Agrícola y Ganadera of the Junta de Andalucía.

As a representative insect species, we used the seed beetle *Callosobruchus maculatus* (Fabricius 1775). Individuals were obtained from a stock population established in 2013 at the Doñana Biological Station (Spanish National Research Council, Seville, Spain), using over 450 founding individuals from an outbred population originating in Southern India (Alexander et al., 2025; Iglesias-Carrasco et al., 2024). This stock population is representative of the phenotypic and genetic variability of the species (Canal et al., 2022; Rodriguez-Exposito and Garcia-Gonzalez, 2021). The beetles used in our study were maintained in 1.5 ml tubes in climate-controlled chambers at 29°C, 40% relative humidity, on a 12 h:12 h dark:light cycle (Alexander et al., 2025; Iglesias-Carrasco et al., 2024). They were cultured on organic mung beans (*Vigna radiata*), simulating semi-natural conditions. In this species, the larval stage is completed inside the beans, and adults emerge to live externally until death (Kalpna et al., 2022). For each sample, groups of 10 adults (sex ratio 1:1), morphologically sexed based on external dimorphic traits, were pooled to obtain the minimum volume required for oxidative stress assays. Adults were euthanised 1–3 days post-emergence by mechanical disruption, achieved by firm compression inside a centrifuge tube. Samples were immediately stored under different conditions (see below).

We used *Mus musculus* Linnaeus 1758 as a representative mammal species. Animals were obtained from Centro de Experimentación Animal Óscar Pintado (CITIUS III, Universidad de Sevilla). Six males C57/J FNT 19-25/2 and four males C57/J FNT 4-10/3 were maintained under 300–315 lx measured 1 m above the soil, on a 12 h:12 h light:dark cycle, at 22±2°C and 65±5% humidity. Mice were maintained in ventilated racks until week four, and then in open racks with enrichment of wood sticks and crinkled paper. The food regime was *ad libitum*, composed of 2018 Teklad Global 18% Protein Rodent Diet for the breeding diet, and 2914 Teklad Irradiated Global 14% Protein Rodent Maintenance Diet until week four after weaning. All mice were euthanised by cervical dislocation on 16 May 2024 (81–87 days old), and muscle and liver samples were collected and assigned to storage treatments (see below). procedures were carried out under ethical approval 16/03/2023/004, granted by the Dirección General de la Producción Agrícola y Ganadera of the Junta de Andalucía.

We used turkeys *Meleagris gallopavo* Linnaeus 1758 as a representative bird species. Once hatched, animals were transported from the hatchery to rearing facilities, where a 10–15 cm layer of disinfected absorbent material was uniformly distributed over the floor (Cano Vilchez, 2020). Poultry houses were preheated to ∼38°C prior to chick placement. Throughout the rearing period, feed and water requirements were monitored to ensure adequacy. Ventilation was maintained to keep carbon dioxide concentrations below 3000 ppm, with temperature and relative humidity maintained below 50% until 21 days of age, and not exceeding 55% until day 28. At 28 days, birds were transferred to fattening units using low-stress transport. Houses were preheated to >25°C, with high light intensity and *ad libitum* access to food and water. Diet formulation was adjusted according to the physiological and nutritional requirements associated with the sex and age of the birds. At the end of the fattening period (i.e. when animals were 120 days old), 10 male birds were transported to the slaughterhouse, where they were rendered unconscious by exposure to controlled concentrations of CO_2_, followed by exsanguination via neck cutting. We then sampled muscle and liver tissues and assigned the samples to storage treatments (see below). All husbandry and slaughter procedures were carried out entirely by PROCAVI S.L. under their routine veterinary oversight following standard industrial protocols. No experimental manipulations were performed on live birds by the research team, and thus no additional animal ethics approval was required.

For all species, muscle samples corresponded to the musculature surrounding the quadriceps. In mice, quadriceps tissue was supplemented with abdominal muscle to obtain sufficient material for all assays. Liver and muscle were finely minced and subdivided so that equivalent portions from each individual were randomly allocated to the different storage treatments, ensuring that any within-tissue heterogeneity was evenly distributed across experimental conditions.

### Temperature and storage conditions

**Fig. 1.**
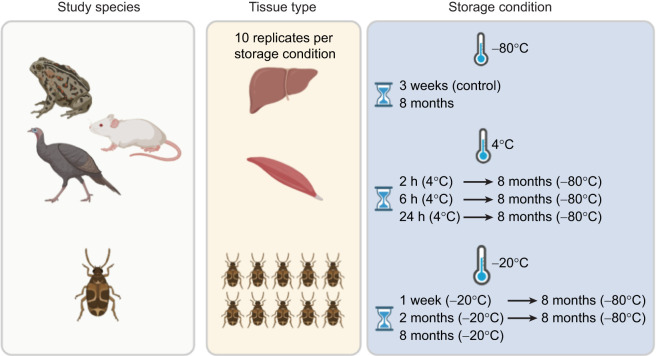
**Overview of the experimental design.** Left: species used in this study: *Pelobates cultripes* (amphibian), *Mus musculus* (mammal), *Meleagris gallopavo* (bird), *Callosobruchus maculatus* (insect). Middle: sample types used in the study (10 replicates per condition). Liver and muscle tissues were obtained from amphibian, mammal and bird samples; whole-body homogenates of 10 pooled individuals were used for insects. Right: summary of storage treatments. Samples were subjected to a range of storage conditions varying in temperature (−80°C, 4°C, −20°C) and duration. Control samples were snap-frozen immediately after dissection and stored at −80°C until analysis 3 weeks after sample collection. Additional treatments included: (i) snap-freezing then storage at −80°C for 8 months until assayed, (ii) storage at 4°C for 2, 6 or 24 h followed by storage at −80°C until assayed 8 months later, (iii) storage at −20°C for 1 week or 2 months followed by storage at −80°C until assayed, or storage at −20°C throughout the experiment until assayed (i.e. 8 months). For mammal and bird samples, those assigned to −20°C treatments were initially snap-frozen in liquid nitrogen during transportation (∼1 h) before storage at −20°C at the Doñana Biological Station (Seville, Spain).

Fig. 1 shows a graphical scheme of the sampling design. Samples (i.e. liver and muscle tissues from amphibian, mammal and bird samples, and whole individuals from insect samples) were assigned to one of eight different storage conditions, with slight procedure variation for mammal and bird samples (see below). Storage conditions included three different temperatures following dissection (4, −20 and −80°C), combined with varying exposure durations (Fig. 1). The sample size was 10 for each storage condition and tissue. For control conditions, a subset of samples was immediately snap-frozen and stored at −80°C until assayed 3 weeks later. Other subsets of samples were first stored at 4°C for 2, 6 or 24 h, then placed at −80°C until assayed 8 months later. Two other subsets of samples were first stored at −20°C for 1 week or 2 months, then placed at −80°C until assayed 8 months later. Finally, two subsets of samples were stored at −20°C and −80°C until assayed 8 months later. For logistical reasons, mammal and bird samples assigned to −20°C storage conditions were first snap-frozen in liquid nitrogen after collection and during transportation (for ∼1 h), then stored at −20°C at the Doñana Biological Station (Seville, Spain). Because of an issue that affected sample integrity, bird samples assigned to the 2 month storage condition at −20°C were excluded from the study. All samples were stored in 1.5 ml microtubes until assayed.

### Oxidative stress assays

To assess oxidative stress and related metabolic status in our study organisms, we quantified key biochemical markers, the activity of three antioxidant enzymes and a marker of lipid damage. We also measured triglyceride levels to test whether these can bias lipid peroxidation measurements.

Tissues were homogenised using a Micra D-1 homogeniser (UHS-RS Technology) with 1:4 w/v of buffer containing 100 mmol l^−1^ Tris-HCl, pH 7.8, 0.1 mmol l^−1^ EDTA and 0.1% Triton X-100. The homogenates were then centrifuged at 14,000 rpm for 30 min at 4°C, and the supernatants were aliquoted in 500 µl microtubes and stored at −80°C until assayed. Throughout the entire process, samples were kept on ice to maintain cold conditions. First, we measured total protein content (needed for standardising antioxidant activity estimates) for each individual using a COBAS INTEGRA Total Protein Gen.2 (TP2) ID test 0-227 system.

GR activity is a key biomarker of cellular recovery capacity and can indicate a compensatory response to increasing oxidative stress. GR activity is also linked to nutritional status, as it depends on the availability of NADPH, a cofactor primarily generated through the pentose phosphate pathway (Halliwell, 2007; Wheeler et al., 1990). GR activity was determined by measuring the decrease in light absorbance at 340 nm due to the oxidation of NADPH to NADP^+^ during the reduction of oxidised glutathione (GSSG) to reduce glutathione (GSH) catalysed by GR. The assay was adapted to the COBAS INTEGRA 400 system following Cribb et al. (1989). Reactions were performed in a phosphate buffer (0.25 mol l^−1^, pH 7.3) containing EDTA (0.5 mmol l^−1^) and NADPH (0.17 mmol l^−1^) diluted in ultrapure water. Calibration and quality control were carried out using RANDOX calibrators, and reagents were prepared in-house following the manufacturer's specifications to ensure equivalent composition and performance. Intra-assay coefficient of variation (CV) was 12.90%.

The enzyme SOD catalyses the dismutation of superoxide radical into oxygen and hydrogen peroxide; this is why it is one of the most important antioxidant defences in cells exposed to oxygen (Halliwell, 2007). SOD activity serves as a reliable biomarker of an animal’s overall physiological condition and its potential for long-term homeostasis and survival, indicating a strong compensatory response to environmental stressors (Costantini, 2019; Halliwell, 2007). Our method to determine SOD activity (Woolliams et al., 1983) uses xanthine and xanthine oxidase, producing superoxide radicals, which react with 2-(4-iodophenil)-3-(4-nitrophenol)-5-pheniltetrazolium chlorate (INT), finally producing formazan, a product with a red colour. SOD activity is measured as the degree of inhibition of the reaction, where one SOD unit is defined as producing 50% inhibition of the INT reduction index under the assay conditions. The method was adapted to the COBAS INTEGRA 400 system, and we used Ransod reagent (Randox Laboratories) consisting of 0.05 mmol l^−1^ xanthine, 0.025 mmol l^−1^ INT in buffer (40 mmol l^−1^ CAPS, pH 7.2, 0.94 EDTA) and 80 U l^−1^ xanthine oxidase. Assays were calibrated and controlled using controls, calibrators and reagents from Randox Laboratories. Intra-assay CV was 11.80%.

The enzyme GPX catalyses the reduction of organic peroxides using glutathione as a substrate, producing water and corresponding alcohols. As a key component of the antioxidant defence system, GPX provides specific insights into an animal's ability to protect itself from lipid peroxidation, a major threat to cellular integrity (Halliwell, 2007). The method used is based on that of Paglia and Valentine (1967), with minor modifications to adapt it to the COBAS INTEGRA 400 system. GPX activity was indirectly measured in an assay in which GSH was oxidised to GSSG by GPX in the presence of cumene hydroperoxide. In this reaction, GSSG is then immediately reduced back to GSH by GR and NADPH, with the concomitant oxidation of NADPH to NADP^+^. GPX activity was determined by monitoring the decrease in light absorbance at 340 nm due to NADPH oxidation. The assay was performed in phosphate buffer (0.05 mol l^−1^, pH 7.2) containing EDTA (4.3 mmol l^−1^), with 4 mmol l^−1^ GSH, 0.34 mmol l^−1^ NADPH and GR ≥0.5 U l^−1^. Cumene hydroperoxide (0.18 mmol l^−1^) was dissolved in 0.9% NaCl, and reactions were stirred in the dark for 30 min. Calibration and quality control were performed using Randox Laboratories calibrators and controls, and reagents were prepared in-house following the manufacturer's specifications to ensure equivalent composition and performance. Intra-assay CV was 3.19%.

Lipid peroxidation is the oxidative damage caused by ROS on fatty acids. As a product of this process, MDA is generated through the peroxidation cascade (Halliwell, 2007). Lipid peroxidation can compromise the fluidity and integrity of cell membranes, disrupting different cellular functions such as ion transport and signal transduction. It can therefore reflect cellular damage from a wide range of environmental stressors and is often associated with fitness-related traits (Costantini, 2008; Metcalfe and Alonso-Alvarez, 2010). In the assay used here, MDA reacts with thiobarbituric acid (TBA) to form red/pink chromogenic products that can be measured as absorbance at 535 nm. Lipid peroxidation was evaluated following the method of Buege and Aust (1978) using a buffer containing HCl (0.25 mol l^−1^), trichloroacetic acid (TCA, 15% w/v) and TBA (0.375% w/v). For each sample, 100 µl of the sample was mixed with 500 µl of HCl-TCA-TBA buffer, vortexed and then incubated at 100°C for 15 min. Samples were subsequently centrifuged at 10,000 rpm for 10 min at 10°C. A 100 µl aliquot of the supernatant was transferred to a 96-well microplate, and absorbance was measured at 535 nm. A 0.1 mmol l^−1^ MDA-bis solution was used for calibration, with serial dilutions ranging from 30 µmol l^−1^ to 1 µmol l^−1^ treated identically to the samples. Intra-assay CV was 7.98%.

Triglycerides are a crucial component of lipid metabolism and energy balance, both of which are intimately linked to an animal stress response and susceptibility to oxidative damage (Remage-Healey and Romero, 2001). Triglyceride concentration was measured using the COBAS INTEGRA Triglycerides (TRIGL) Test TRIGL, ID test 0-010. Intra-assay CV was 1.6%.

### Statistical analysis

All analyses were conducted in R (version 4.2.3). Before model fitting, we assessed parametric assumptions using Levene's (*car* package) and Barlett's tests for homocedasticity and normality of residuals, respectively. As we treated samples from each species as independent, we built separate linear models for each species (amphibian, insect, mammal, bird) and the different response variables (SOD, GR and GPX activity, and MDA concentration). The models included the storage condition and tissue type (except for beetles) as fixed effects, and individual as the random factor (except for beetles). To test for differences across storage conditions, we calculated estimated marginal means, and pairwise comparisons adjusted for false discovery rate were conducted with the help of the package *emmeans*. In all cases, we compared the effect of the different storage conditions against the control group, i.e. samples snap-frozen using liquid nitrogen, then stored at −80°C until assayed 3 weeks later. For graphical comparison, treatment effects were expressed relative to the control by centring the estimated marginal means (EMMs) obtained from the models. For each taxon (and tissue when applicable), we extracted the EMMs for all storage treatments and subtracted the EMM of the control group. This procedure provides the deviation of each treatment from the control on the original response scale and was applied for visualisation in the figures. We also plotted absolute values of each parameter across treatments (see below).

We additionally evaluated whether variation in lipid peroxidation was associated with triglyceride levels by fitting models separately for each taxon. In these analyses, MDA levels were included as the response variable, while storage condition and, when applicable, tissue type (and their interaction) were included as fixed factors. triglyceride level was included as an additive covariate and individual was included as the random factor (except for beetles). Both MDA and triglyceride level were log-transformed prior to analysis to meet parametric assumptions.

## RESULTS AND DISCUSSION

### Effect of storage conditions across tissue type and taxa

**Fig. 2.**
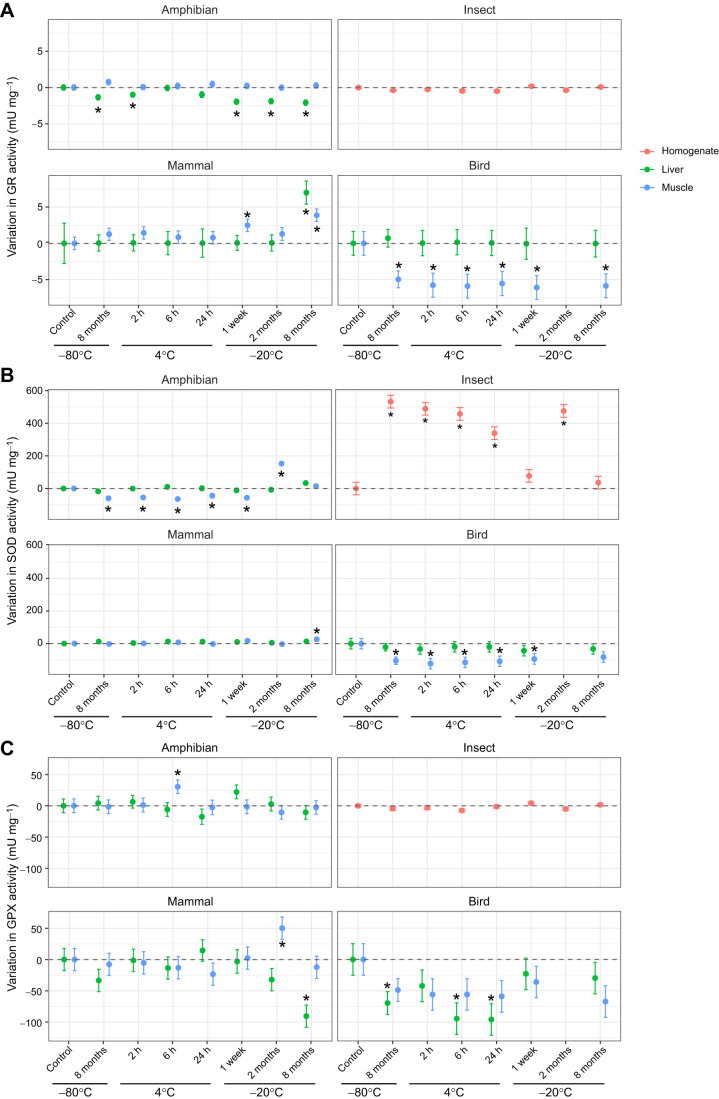
**Changes in antioxidant enzyme activity across treatments.** Changes in (A) glutathione reductase (GR), (B) superoxide dismutase (SOD) and (C) glutathione peroxidase (GPX) activity, centred relative to the control condition (snap-freezing followed by storage at −80°C for 3 weeks before analysis; dashed line at zero). Estimated mean effects (points) and s.e.m. (bars) are presented for each combination of storage temperature and duration for liver (green) and muscle (blue) tissues for the representative amphibian (*Pelobates cultripes*), bird (*Meleagris gallopavo*) and mammal (*Mus musculus*), and whole-body homogenate (red) for the representative insect (*Callosobruchus maculatus*). Asterisks denote treatments that differ significantly from the control condition. Corresponding *post hoc* results are reported in Tables S4, S6 and S8.

**Fig. 3.**
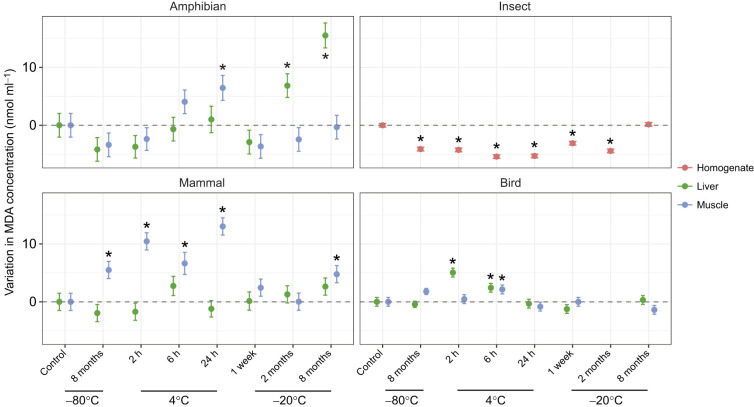
**Lipid damage across treatments.** Changes in the concentration of malondialdehyde (MDA) as a measure of lipid peroxidation across storage treatments, centred relative to the control condition (snap-freezing followed by storage at −80°C for 3 weeks before analysis; dashed line at zero). Estimated effects (points) and s.e.m. (bars) are shown for each combined treatment of temperature and duration for liver (green) and muscle (blue) tissues of the representative amphibian (*P. cultripes*), bird (*M. gallopavo*) and mammal (*M. musculus*), and whole-body homogenate (red) for the insect (*C. maculatus*). Asterisks indicate treatments that differ significantly from the control condition. *Post hoc* results are provided in Table S8.

In the amphibian samples, the interaction between storage conditions and tissue type significantly influenced total protein concentration (Table S1). *Post hoc* contrasts indicated only limited differences from the control in the muscle, with a significant increase detected in samples stored at 4°C for 6 h, and a significant decrease in samples stored at −20°C for 2 months (Table S2, Figs S1 and S2). Storage conditions also influenced the activity of amphibian antioxidant enzymes across tissues. While GR activity did not vary across storage conditions in the muscle, it decreased significantly in the liver upon long-term storage at −80°C, after 2 h at 4°C, or due to storage at −20°C regardless of the storage duration (Fig. 2A; Fig. S3A, Tables S3 and S4). In contrast, SOD activity was affected by storage conditions only in the muscle, generally leading to reductions across treatments, except in samples stored at −20°C for 2 months (Fig. 2B; Fig. S3B, Tables S5 and S6). GPX activity was only subtly affected by storage conditions: we only detected significant increases in enzymatic activity in muscle samples stored at 4°C for 6 h (Fig. 2C; Fig. S3C, Tables S7 and S8). Regarding lipid peroxidation (MDA levels), we observed significantly higher values in liver samples stored at −20°C for 2 or 8 months, and in muscle samples stored at 4°C for 24 h (Fig. 3; Fig. S4, Tables S9 and S10).

In the insect samples, total protein concentration varied strongly across storage conditions (Table S1), with all treatments showing significantly lower protein levels than the control condition (Table S2, Figs S1 and S2). In contrast, whole-body GR activity was not affected by storage conditions (Fig. 2A; Fig. S3A, Tables S3 and S4). Whole-body SOD activity was significantly higher compared with control conditions across all treatments except in samples stored at −20°C for 1 week and 8 months (Fig. 2B; Fig. S3B, Tables S5 and S6). Storage treatment had no effect on beetle GPX activity (Fig. 2C; Fig. S3C, Tables S7 and S8). Lipid peroxidation significantly decreased across all treatments compared with control conditions, except in samples stored at −20°C for 8 months (Fig 3; Fig. S4, Tables S9 and S10).

In the mammal samples, total protein concentration differed significantly across storage conditions and between tissues, though their interaction was not significant (Table S1). Several treatments reduced liver protein concentration relative to the control, whereas protein levels in muscle remained unchanged (Table S2, Fig. S1 and S2). Storage conditions did not largely affect GR activity across tissues, except in samples stored at −20°C for 8 months in both tissues, and those at −20°C for 1 week in muscle (Fig. 2A; Tables S3 and S4). Likewise, SOD activity only showed increases when muscle samples were stored at −20°C for 8 months (Fig. 2B; Fig. S3B, Tables S5 and S6). The effect of storage conditions on GPX activity was contrasting across tissues: while muscle samples stored at −20°C for 2 months showed higher activity, liver samples stored at the same temperature for 8 months experienced lower GPX activity (Fig. 2C; Fig. S3C, Tables S7 and S8). Lipid peroxidation was unaffected by storage conditions in the liver, but it significantly increased in muscle samples stored at −80°C and −20°C for 8 months, and at 4°C for 2, 6 or 24 h (Fig. 3; Fig. S4, Tables S9 and S10).

In bird samples, total protein concentration was significantly influenced by the interaction between storage conditions and tissue type (Table S1). Liver protein content increased in samples stored at −20°C for 1 week and 8 months, whereas muscle protein concentration did not differ from control values (Table S2, Figs S2 and S3). GR activity was not significantly influenced by storage conditions in the liver (Table S3), but it decreased across all treatments in muscle (Fig. 2A; Fig. S3A, Tables S3 and S4). Likewise, SOD activity was not affected by storage condition in the liver but showed decreases across treatments in muscle samples (Fig. 2B; Fig. S3B, Tables S5 and S6), although it was not significant in samples stored at -80 °C for 8 months. GPX activity was only affected by storage conditions in the liver in samples stored at −80°C for 8 months, and at 4°C for 6 and 24 h (Fig. 2C; Fig. S3C, Tables S7 and S8). Lipid peroxidation was significantly higher in liver samples stored at 4°C for 2 and 6 h, whereas storage conditions influence lipid peroxidation in muscle samples only at 4°C for 6 h (Fig. 3; Fig. S4, Tables S9 and S10).

**Fig. 4.**
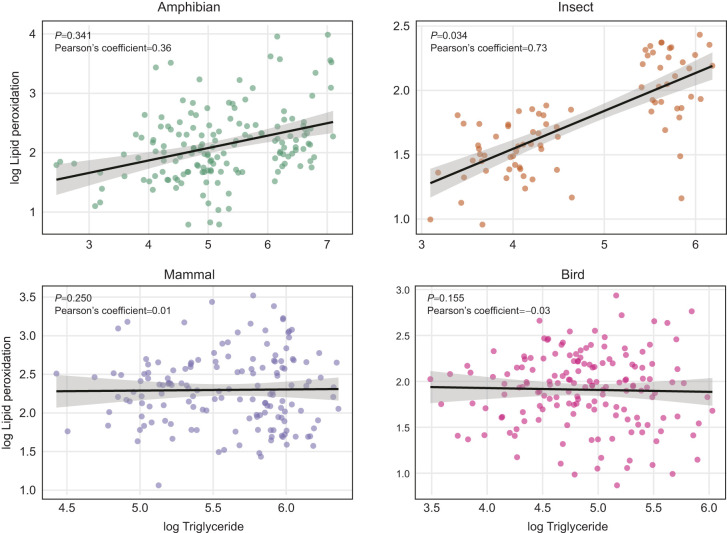
**Relationship between triglyceride levels and MDA concentration across storage treatments.** MDA (as a measure of lipid peroxidation; nmol ml^−1^) and triglyceride levels (mg dl^−1^) for the representative amphibian (*P. cultripes*), insect (*C. maculatus*), mammal (*M. musculus*) and bird (*M. gallopavo*) (log transformed). Lines represent linear model fits with 95% confidence intervals. For the insect, the two visually distinct point clusters reflect treatment-induced variation in triglyceride values rather than an underlying biological grouping (see Results and Fig. S7). *P*-values and Pearson's correlation coefficients are reported in each panel.

We further evaluated whether variation in MDA was influenced by triglyceride levels after accounting for the effects of storage conditions and tissue type. In amphibian samples, triglyceride levels were not significantly associated with MDA (χ_1,157_=0.90; *P*=0.342, Pearson's coefficient=0.36; Fig. 4). In contrast, triglyceride levels were significantly associated with MDA in insects (χ_1,80_=9.20, *P*=0.034; Pearson's coefficient=0.73; Fig. 4). The two visually distinct clusters observed in insect samples (Fig. 4) reflect differences in triglyceride concentration across treatments (Figs S5–S7). However, these patterns do not consistently align with storage severity, as some control and long-term storage treatments exhibited similarly high triglyceride values. At present, we are unable to unambiguously identify the cause of this variation. In mammal and bird samples, triglyceride levels were not significantly associated with MDA (χ_1,157_=1.32, *P*=0.250 and χ_1,157_=2.02; *P*=0.155, respectively; Fig. 4). Changes in triglyceride concentration across treatments and tissues are reported in Tables S11 and S12, and Figs S5 and S6.

### Key findings and implications

Our study highlights the effect that storage conditions may have on the stability of oxidative stress biomarkers. We found that both the magnitude and direction of the different measurements varied depending on the specific biomarker, tissue type and species. We also observed that the putative confounding effect of lipid concentration on lipid peroxidation quantification could also be taxon dependent. Overall, our findings underscore the importance of considering methodological constraints when designing studies involving oxidative stress markers, with an emphasis on longitudinal and field-based approaches. Suboptimal storage conditions may bias oxidative stress measurements and potentially lead to flawed mechanistic interpretations regarding the role of redox pathways in eco-evolutionary processes.

Ecological and evolutionary studies, whether experimental or conducted in the field, often face methodological challenges related to sample collection and preservation (Pyke and Ehrlich, 2010; Vaught and Henderson, 2011). In many cases, researchers must collect and process biological samples under suboptimal conditions, particularly when working in remote environments or when handling large numbers of individuals over extended periods of time. One major concern is the stability of oxidative stress markers, which can be sensitive to the temperature and duration of storage before analysis. Our results show that both enzymatic activity and lipid peroxidation can be significantly altered depending on the method and duration of sample preservation. While immediate snap-freezing remains the gold standard for preserving oxidative stress markers, our results suggest that short-term refrigeration at 4°C, followed by proper long-term freezing may be a viable option in field-based studies for some markers and tissues. In addition, medium-term (i.e. up to 2 months) storage at −20°C can be a practical alternative when cryopreservation is not feasible. However, the suitability of this approach is context dependent and may not be appropriate for all biomarkers, species or tissues, highlighting the need for validation of preservation protocols in each study system.

Longitudinal approaches measuring oxidative stress biomarkers are crucial for understanding the role of oxidative damage in life-history trade-offs, as they provide insights into how organisms allocate resources between maintenance, reproduction and survival over the course of a lifetime (Clutton-Brock and Sheldon, 2010; Stroud and Ratcliff, 2025). In longitudinal studies, measurements are often conducted altogether at the end of the experiment or field campaign. Our results demonstrate that long-term storage can affect the stability of oxidative stress parameters, even when using a temperature considered optimal (i.e. snap-freezing in liquid nitrogen and −80°C until assayed). For instance, long-term (i.e. 8 months) preservation at −80°C resulted in decreased GR and SOD activity in amphibian and bird samples, or increased SOD activity in insect samples. Furthermore, long-term −80°C storage led to marked changes in lipid peroxidation levels in mammal and insect samples. Likewise, long-term storage at −20°C resulted in deviations from baseline levels of different oxidative stress markers, a pattern observed across tissues and species included in our study. These findings highlight that even under ‘optimal’ conditions, long-term storage can introduce biases that obscure biological patterns, complicating the interpretation of oxidative stress dynamics across time. Therefore, longitudinal studies aiming to link oxidative markers to fitness-related traits must account for potential storage effects, ideally by measuring the parameters as soon as possible after sample collection, or validating marker stability across storage duration or standardising times to analysis post-collection.

To assess oxidative stress variation across tissues or species, it is crucial to use comparable measurements of redox pathways. In this line, our study shows that the stability of oxidative stress markers in response to storage conditions is variable across tissues and species. Amphibian and bird samples exhibited the most pronounced changes in enzymatic activity, particularly in GR and SOD activity. In insect samples, whole-body enzymatic activity of markers such as GPX and GR appeared more stable under suboptimal storage conditions, whereas SOD showed a marked decline across treatments. Mammals generally experienced minimal enzymatic changes under suboptimal storage. Lipid peroxidation (MDA) increased sharply across in amphibian, mammal and bird samples, probably reflecting the accumulation of aldehyde as cellular membranes degrade during storage (Halliwell and Whiteman, 2004; Tsikas, 2017). Insects (beetles) were the only group in which MDA consistently decreased under suboptimal storage conditions, which could be explained by the formation of MDA–protein adducts, as reactive aldehydes such as MDA readily bind covalently to proteins (Grintzalis et al., 2013; Mateos et al., 2005), thereby reducing the pool of free detectable MDA. Unfortunately, our protocol did not include a step to release protein-bound MDA prior to quantification. These findings emphasise that storage effects are taxon specific and underscore the importance of carefully selecting and standardising storage protocols when interpreting oxidative stress biomarkers in ecological and evolutionary research.

Although we expected antioxidant enzyme activity to decrease with time and temperature, our results show that storage effects did not always follow these predictions. The observed increases in enzymatic activity may stem from oxidative stress during storage or thawing, which can release or generate additional substrates and disrupt cellular structures, artificially elevating measurable enzyme activity (Hartmann and Asch, 2019; Kang et al., 2014). Such storage-driven increases in enzymatic activity could confound biological interpretations, particularly in comparative studies aiming to detect subtle physiological differences among treatment groups or populations. Moreover, our findings align with previous work in avian blood, showing that the interpretation of changes in lipid peroxidation markers such as MDA can be influenced by the intrinsic lipid content of the sample (Pérez-Rodríguez et al., 2015; Romero-Haro et al., 2015). As MDA is a product of lipid degradation, its levels are shaped not only by oxidative challenge but also by the absolute abundance of peroxidisable substrates (Halliwell and Lee, 2010; Pérez-Rodríguez et al., 2015; Romero-Haro et al., 2015; Sies et al., 2005; Wallace et al., 2010). In our study, we observed in insect samples a positive relationship between MDA levels and triglyceride concentration, supporting the inclusion of lipid concentration as a covariate when measuring lipid peroxidation, which seems to be taxon specific. This also reinforces the idea of understanding the biology of oxidative stress parameters to avoid misinterpretation, specially concerning potential trade-offs between life-history traits and oxidative stress dynamics.

### Methodological considerations in eco-evolutionary research

Several methodological limitations and considerations are important to acknowledge when interpreting oxidative stress measurements in ecological and evolutionary studies. Variability between laboratory sessions conducted months or years apart may introduce additional sources of analytical noise, as changes in calibration, reagent batches, instrumentation or ambient conditions may confound biological patterns and obscure distinctions between sample age, treatment effects or organismal traits. To minimise such issues, researchers are encouraged to analyse samples from all treatment groups within the same analytical session whenever feasible, randomise samples from different treatments across sessions, and statistically account for batch effects when unavoidable. Moreover, because longitudinal studies linking oxidative markers to fitness-related traits may be particularly sensitive to storage artefacts, parameters should ideally be measured as soon as possible after sample collection or, when this is not feasible, marker stability across storage duration should be explicitly validated. Furthermore, avoiding bias requires careful experimental design, including balanced sampling across treatments, ensuring comparable storage durations and excluding samples with uncertain preservation histories. Reproducibility in oxidative stress research depends critically on transparent reporting of tissue identity and handling details. Different tissues vary in biochemical composition, enzymatic content and susceptibility to oxidation or degradation, making cross-tissue comparisons essential for understanding redox and metabolic responses *in vivo* (Dawson et al., 2025; Young et al., 2013), similar to findings in other stress-linked processes such as telomere dynamics (Burraco et al., 2023a,b, 2025). Specifically, although blood is the most commonly used matrix in vertebrate eco-physiology, its specific sensitivity to storage conditions remains insufficiently characterised; hence, future efforts should explicitly evaluate biomarker stability in this tissue to enhance comparability across studies. Finally, methodological factors such as storage container type, buffer composition, buffer-to-tissue ratios, homogenisation procedures and handling of homogenates prior to assays can further influence marker stability and should therefore be reported consistently.

### Conclusions

Our study provides insights for researchers aiming to incorporate oxidative stress biomarkers into ecological and evolutionary studies, particularly in field-based and longitudinal approaches, where methodological constraints are prevalent. We offer encouraging evidence that measurements of antioxidant enzymatic activity and lipid peroxidation can remain relatively stable under moderate short-term refrigeration, which may be a feasible and practical solution in field settings where immediate snap-freezing is not possible. However, in some cases, we also demonstrate that long-term storage, even under optimal conditions, can significantly alter the values of key biomarkers, potentially introducing biases that may obscure biological patterns or generate spurious conclusions. Finally, our results underscore the importance of evaluating possible confounding factors when measuring oxidative stress markers, to avoid misinterpretations surrounding the association between an organism's life history and its redox status. These findings highlight the need for cautious interpretation of oxidative stress data and emphasise the validation of storage protocols for the species, tissue and biomarker of interest in ecological and evolutionary studies.

## Supplementary Material

10.1242/jexbio.251748_sup1 Supplementary information
