# Supplementary material for: Effects of storage conditions on oxidative stress biomarkers: methodological implications for ecological and evolutionary studies
Source: J Exp Biol. 2026 Mar 5;229(5):jeb251748. doi: 10.1242/jeb.251748 (PMC12989074; doi:10.1242/jeb.251748)
Supplement: Supplementary information [file jexbio-229-251748-s1.pdf]

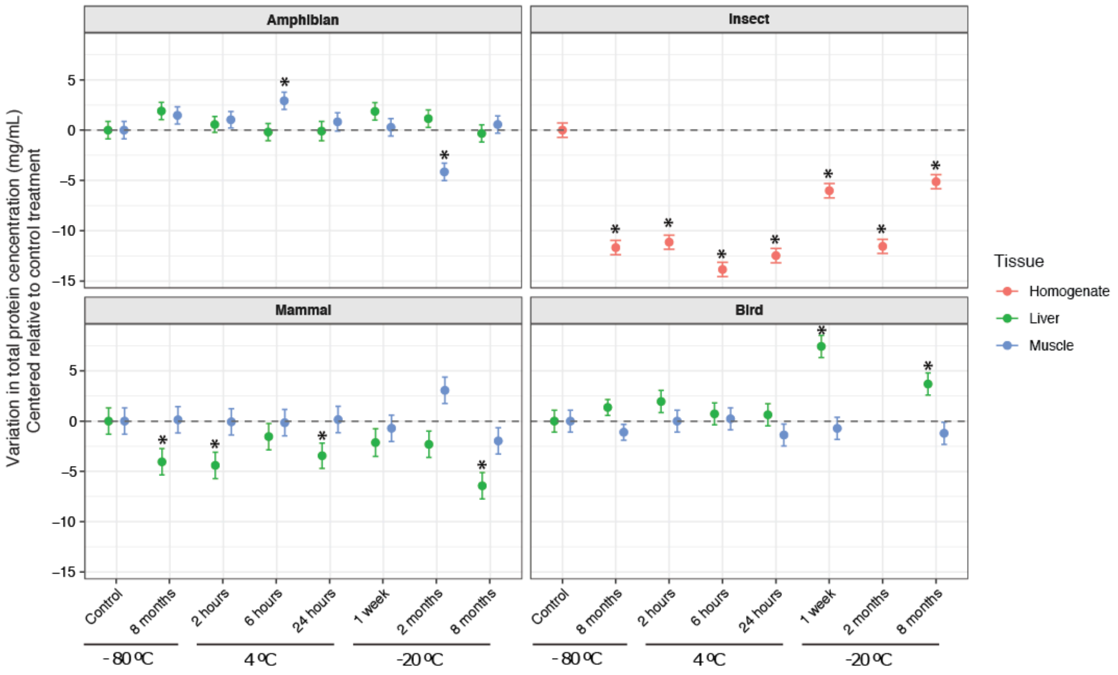

Fig. S1. Changes in total protein concentration across storage treatments (see Methods), centered relative to the control condition, *i.e.,* snap-freezing followed by storage at -80 °C and measurements conducted three weeks after sample collection. Estimated effects (points) and standard errors (bars) are shown for each combined treatment of temperature and time in liver (green) and muscle (blue), except for insects where whole-body samples were used. The dashed horizontal line indicates the control reference value (centered change = 0). Species included are: *Pelobates cultripes* (amphibian, top left), *Callosobruchus maculatus* (insect, top right), *Mus musculutus* (mammal, bottom left), and *Meleagris gallopavo* (bird, bottom right). Asterisks indicate treatments that differ significantly from the control condition. Post-hoc results are provided in Supplementary Table S2.

Journal of Experimental Biology • Supplementary information

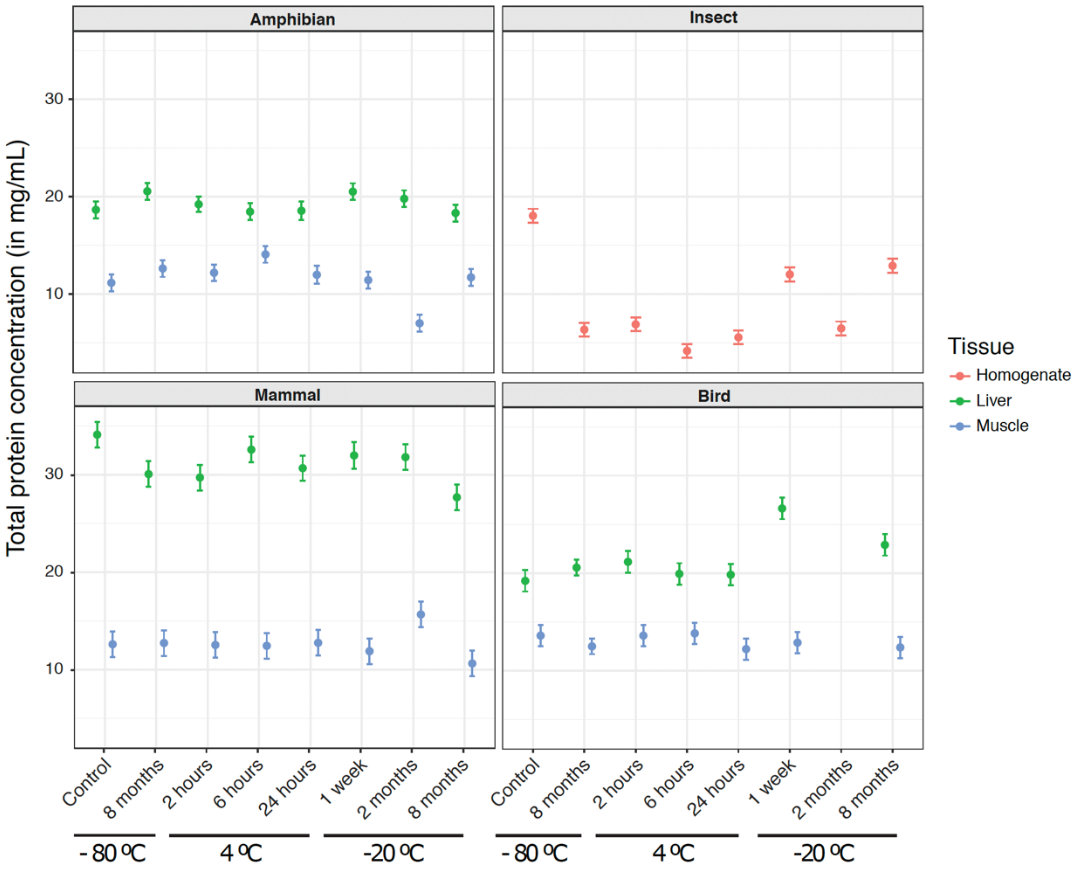

Fig. S2. Changes in the absolute values of total protein concentration across storage treatments (see Methods). Estimated effects (points) and standard errors (bars) are shown for each combined treatment of temperature and time in liver (green) and muscle (blue), except for insects where whole-body samples were used. The dashed horizontal line indicates the control reference value (centered change = 0). Species included are: *Pelobates cultripes* (amphibian, top left), *Callosobruchus maculatus* (insect, top right), *Mus musculutus* (mammal, bottom left), and *Meleagris gallopavo* (bird, bottom right).

Journal of Experimental Biology • Supplementary information

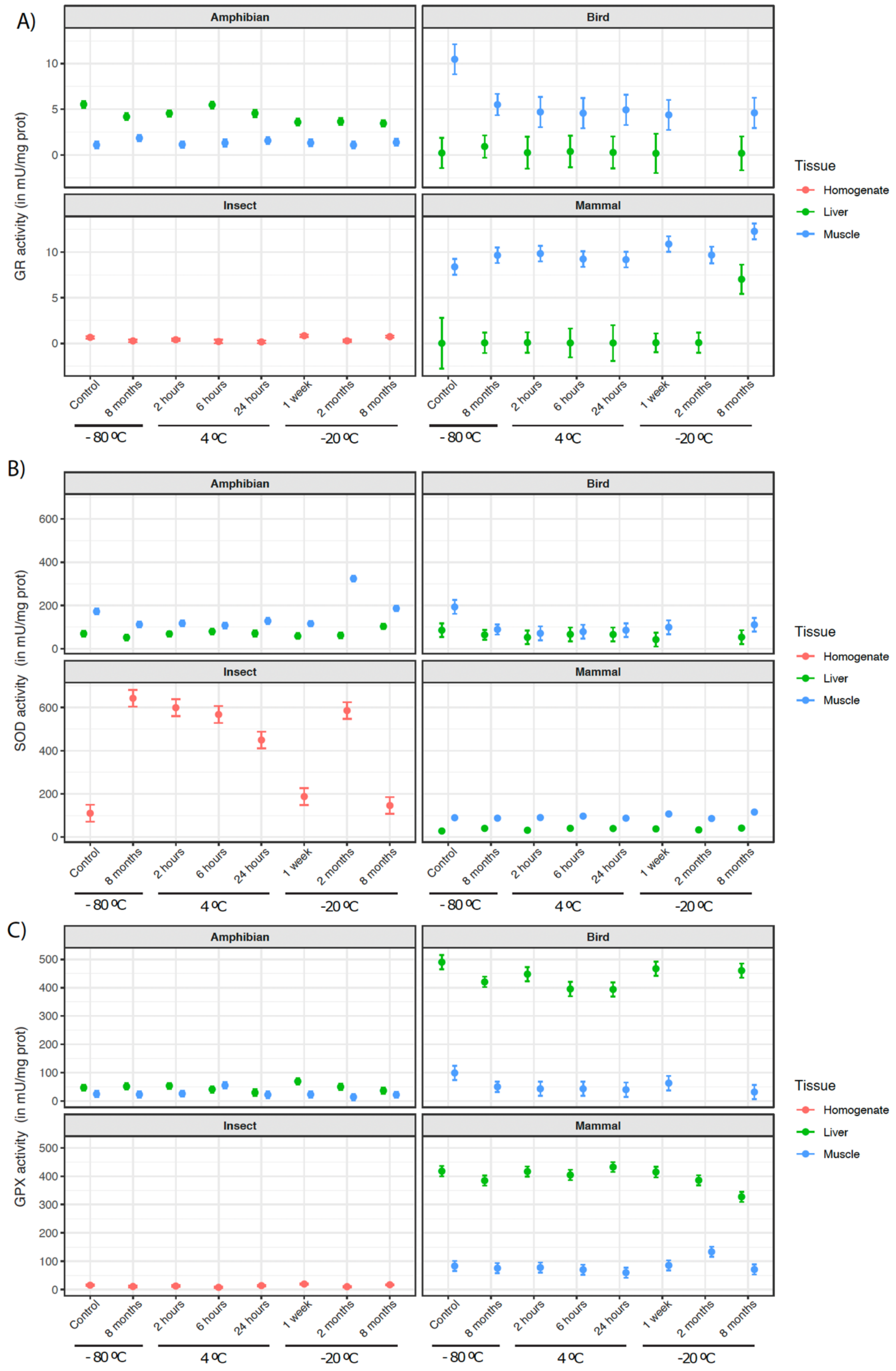

Fig. S3. Changes in the absolute values of glutathione reductase (GR, panel A), superoxide dismutase (SOD, panel B), and glutathione peroxidase (GPX, panel C) across storage treatments (see Methods). Estimated effects (points) and standard errors (bars) are shown for each combined treatment of temperature and time in liver (green) and muscle (blue), except for insects where whole-body samples were used. The dashed horizontal line indicates the control reference value (centered change = 0). Species included are: *Pelobates cultripes* (amphibian, top left), *Callosobruchus maculatus* (insect, top right), *Mus musculutus* (mammal, bottom left), and *Meleagris gallopavo* (bird, bottom right).

Journal of Experimental Biology • Supplementary information

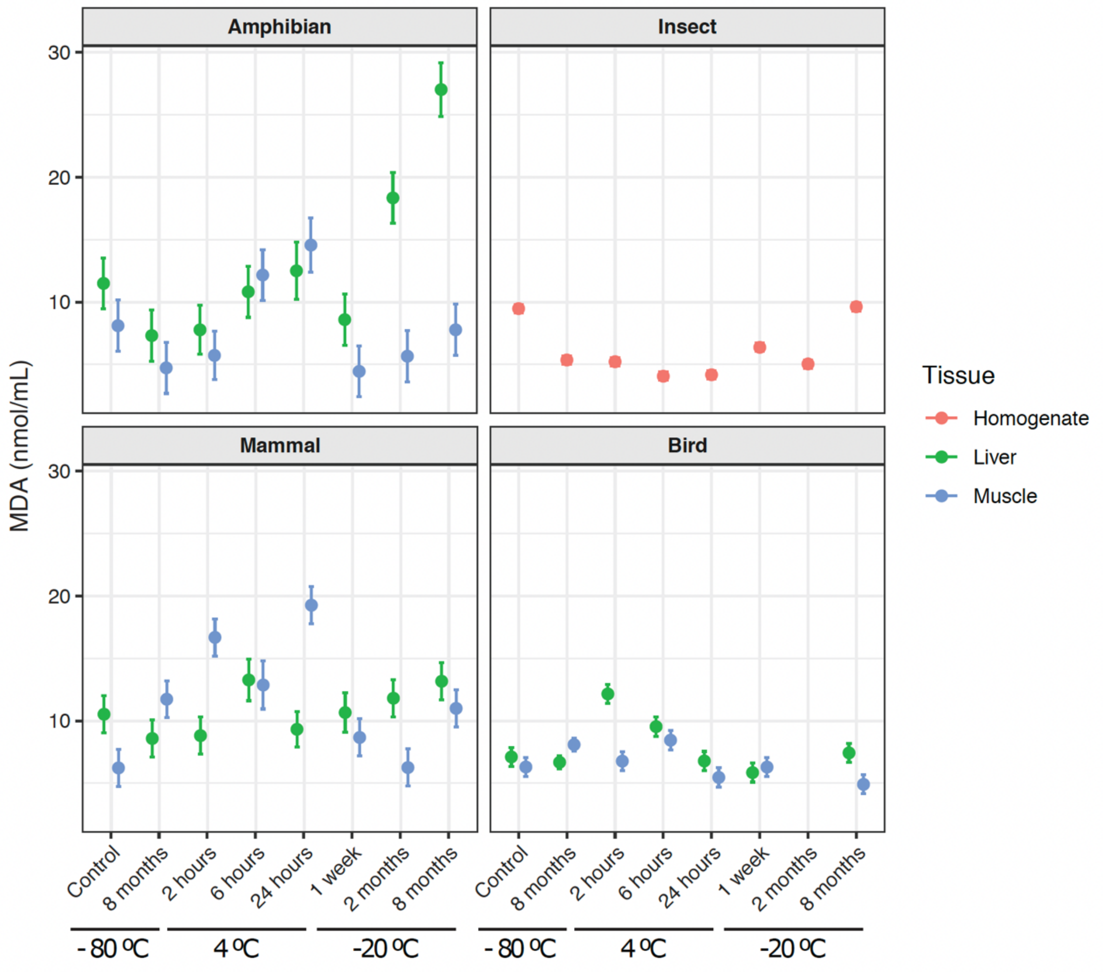

Fig. S4. Changes in the absolute concentration of malondialdehyde (MDA) across storage treatments (see Methods). Estimated effects (points) and standard errors (bars) are shown for each combined treatment of temperature and time in liver (green) and muscle (blue), except for insects where whole-body samples were used. Species included are: *Pelobates cultripes* (amphibian, top left), *Callosobruchus maculatus* (insect, top right), *Mus musculutus* (mammal, bottom left), and *Meleagris gallopavo* (bird, bottom right).

Journal of Experimental Biology • Supplementary information

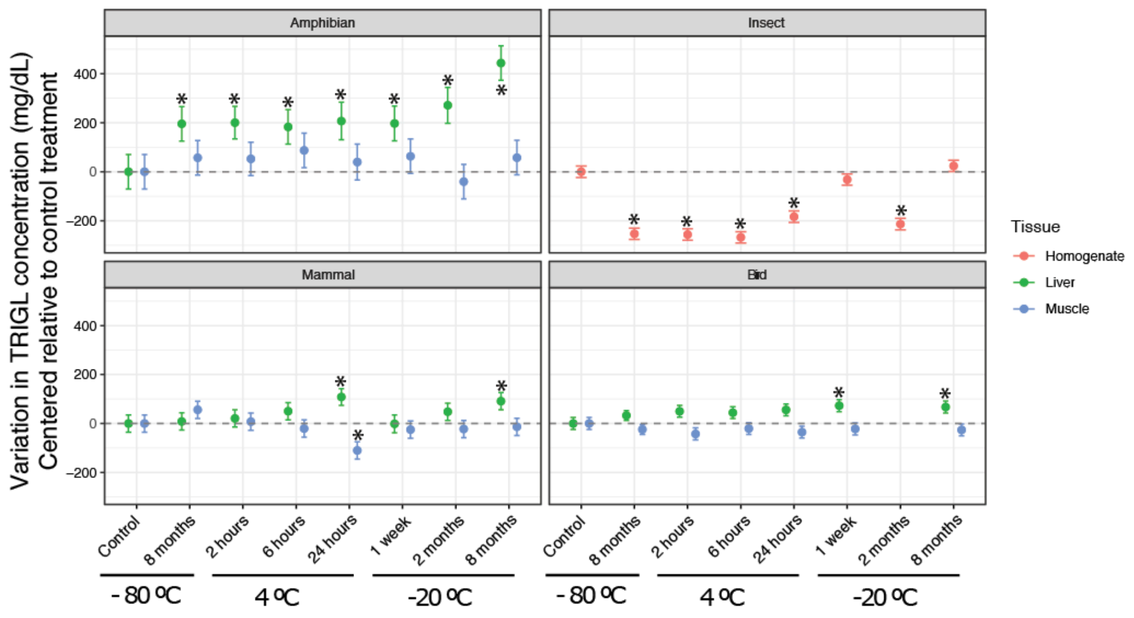

Fig. S5. Changes in the concentration of triglycerides (TRIGL) across storage treatments (see Methods). Estimated effects (points) and standard errors (bars) are shown for each combined treatment of temperature and time in liver (green) and muscle (blue), except for insects where whole-body samples were used. The dashed horizontal line indicates the control reference value (centered change = 0). Species included are: *Pelobates cultripes* (amphibian, top left), *Callosobruchus maculatus* (insect, top right), *Mus musculutus* (mammal, bottom left), and *Meleagris gallopavo* (bird, bottom right).

Journal of Experimental Biology • Supplementary information

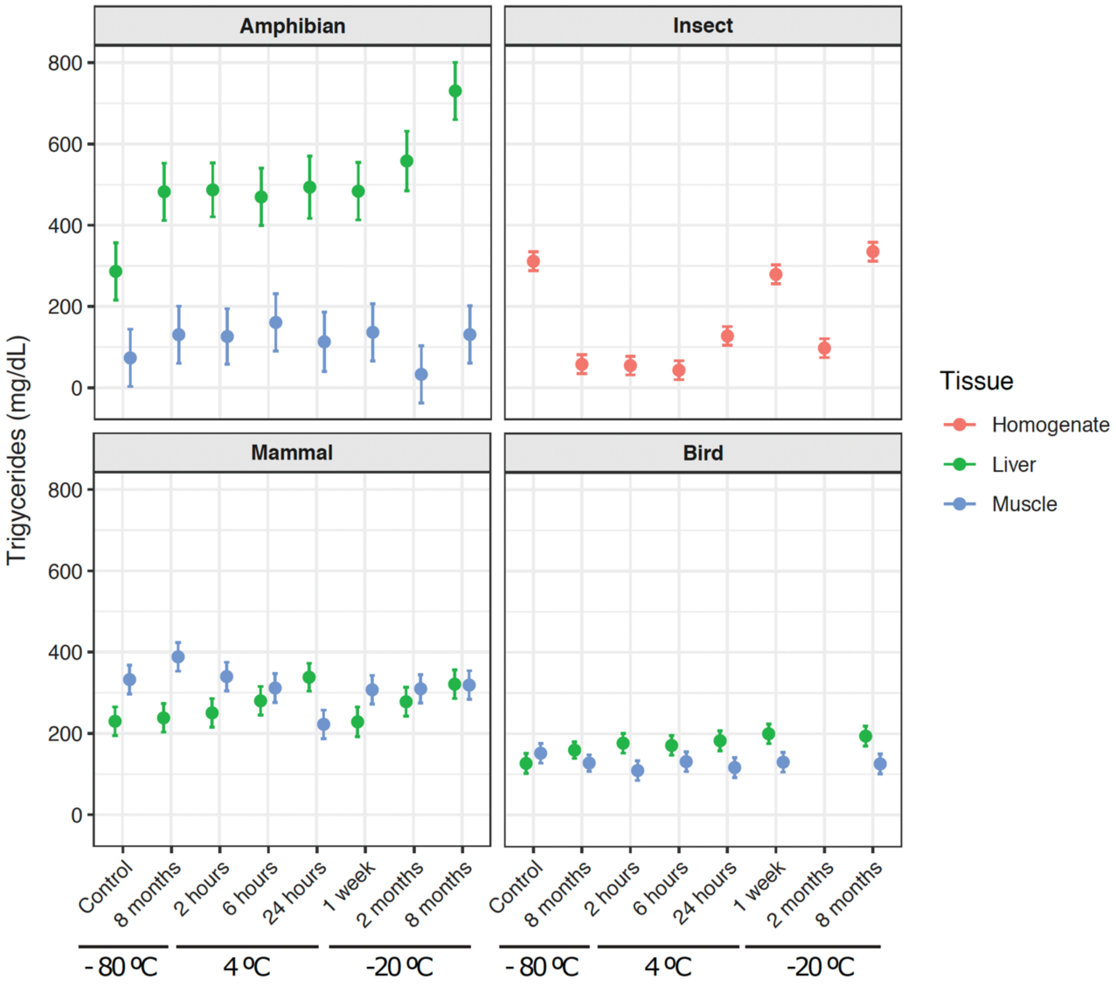

Fig. S6. Changes in the absolute concentration of triglycerides (TRIGL) across storage treatments (see Methods). Estimated effects (points) and standard errors (bars) are shown for each combined treatment of temperature and time in liver (green) and muscle (blue), except for insects where whole-body samples were used. Species included are: *Pelobates cultripes* (amphibian, top left), *Callosobruchus maculatus* (insect, top right), *Mus musculutus* (mammal, bottom left), and *Meleagris gallopavo* (bird, bottom right).

Journal of Experimental Biology • Supplementary information

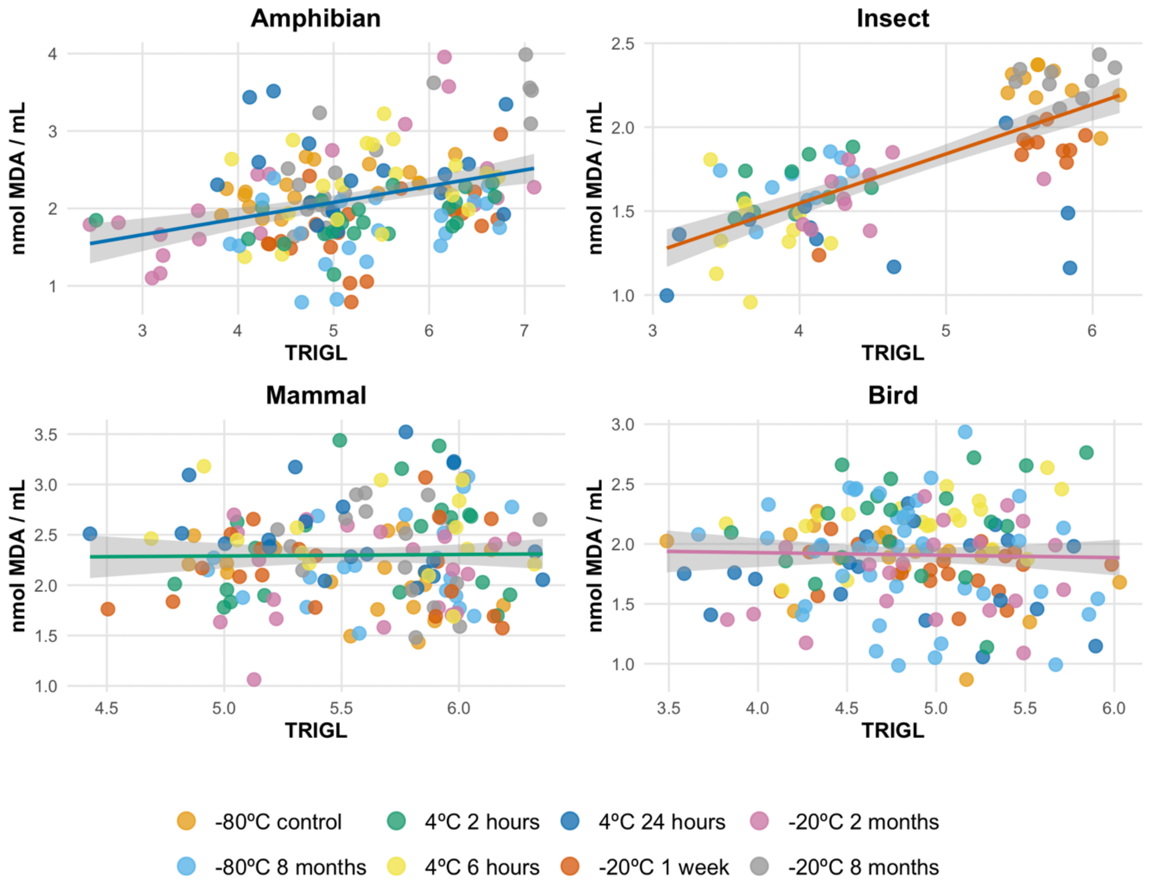

Fig. S7. Relationship between triglyceride levels (TRIGL) and malondialdehyde (MDA) concentration (both log-transformed) across storage treatments. Points are coloured according to storage treatment, and lines represent linear model fits with 95% confidence intervals. Species included are *Pelobates cultripes* (amphibian, top left), *Callosobruchus maculatus* (insect, top right), *Mus musculus* (mammal, bottom left), and *Meleagris gallopavo* (bird, bottom right).

Journal of Experimental Biology • Supplementary information

Table S1. Summary of the linear models (mixed for all species except for beetle samples) testing for the effects of storage conditions, tissue type, and their interaction on the activity of total protein concentration in amphibian, insect, mammal, and bird samples.

**Total protein**	**Amphibian**		**Insect**		**Mammal**		**Bird**	
	**Chi-sq**	**P-value**	**Chi-sq**	**P-value**	**Chi-sq**	**P-value**	**Chi-sq**	**P-value**
**Storage conditions**	$X_{7,153}$ = 18.96	0.008	$X_{7,73}$ = 45.44	<0.001	$X_{7,154}$ = 18.57	0.010	$X_{6,154}$ = 17.03	0.009
**Tissue**	$X_{1,158}$ = 324.31	<0.001			$X_{1,159}$ = 880.49	<0.001	$X_{1,159}$ = 247.92	<0.001
**Storage conditions * Tissue**	$X_{7,153}$ = 28.20	<0.001			$X_{7,153}$ = 8.19	0.316	$X_{6,153}$ = 20.86	0.002

Table S2. Summary of pairwise contrasts comparing different sample preservation conditions against the control condition (i.e., snap freezing in liquid nitrogen, stored at -80ºC and measured three weeks later) for total protein content in amphibian, insect, mammal, and bird samples. For each group, results are presented by tissue type, including the *t*-ratio and the corrected false-discovery-rate *P*-value for each comparison.

**Total Protein contrasts**	**Amphibian**			**Insect**			**Mammal**			**Bird**		
	**Tissue**	**t-ratio**	**P-value**	**Tissue**	**t-ratio**	**P-value**	**Tissue**	**t-ratio**	**P-value**	**Tissue**	**t-ratio**	**P-value**
**-80ºC control vs -80ºC 8 months**	Liver	1.56	0.120	Whole-body	-11.69	**<0.001**	Liver	-2.31	**0.022**	Liver	1.04	0.300
**-80ºC control vs 4ºC 2 hours**	Liver	0.48	0.629	Whole-body	-11.15	**<0.001**	Liver	-2.51	**0.013**	Liver	1.29	0.200
**-80ºC control vs 4ºC 6 hours**	Liver	-0.16	0.876	Whole-body	-13.87	**<0.001**	Liver	-0.88	0.381	Liver	0.48	0.635
**-80ºC control vs 4ºC 24 hours**	Liver	-0.07	0.942	Whole-body	-12.49	**<0.001**	Liver	-2.00	**0.047**	Liver	0.42	0.678
**-80ºC control vs -20ºC 1 week**	Liver	1.53	0.128	Whole-body	-6.02	**<0.001**	Liver	-1.18	0.238	Liver	4.91	**<0.001**
**-80ºC control vs -20ºC 2 months**	Liver	0.94	0.350	Whole-body	-11.57	**<0.001**	Liver	-1.32	0.190			
**-80ºC control vs -20ºC 8 months**	Liver	-0.27	0.786	Whole-body	-5.13	**<0.001**	Liver	-3.67	**<0.001**	Liver	2.44	**0.016**
**-80ºC control vs -80ºC 8 months**	Muscle	1.21	0.228				Muscle	0.07	0.941	Muscle	-0.84	0.401
**-80ºC control vs 4ºC 2 hours**	Muscle	0.87	0.386				Muscle	-0.04	0.968	Muscle	0.01	0.990
**-80ºC control vs 4ºC 6 hours**	Muscle	2.40	**0.018**				Muscle	-0.09	0.927	Muscle	0.15	0.879
**-80ºC control vs 4ºC 24 hours**	Muscle	0.66	0.509				Muscle	0.09	0.932	Muscle	-0.91	0.363
**-80ºC control vs -20ºC 1 week**	Muscle	0.23	0.818				Muscle	-0.41	0.686	Muscle	-0.48	0.635
**-80ºC control vs -20ºC 2 months**	Muscle	-3.42	**0.001**				Muscle	1.75	0.083			
**-80ºC control vs -20ºC 8 months**	Muscle	0.46	0.646				Muscle	-1.12	0.263	Muscle	-0.80	0.425

Table S3. Summary of the linear models (mixed for all species except for beetle samples) testing for the effects of storage conditions, tissue type, and their interaction on the activity of the enzyme glutathione reductase (GR) in amphibian, insect, mammal, and bird samples.

Glutathione reductase	Amphibian		Insect		Mammal		Bird	
	Chi-sq	P-value	Chi-sq	P-value	Chi-sq	P-value	Chi-sq	P-value
**Storage conditions**	$X_{7,152}$ =20.31	**0.005**	$X_{7,63}$ = 3.95	**0.001**	$X_{7,105}$ =24.69	**<0.001**	$X_{6,143}$ =5.33	0.502
**Tissue**	$X_{1,158}$ = 317.38	**<0.001**			$X_{1,112}$ = 260.68	**<0.001**	$X_{1,148}$ = 37.27	**<0.001**
**Storage conditions * Tissue**	$X_{7,152}$ = 24.16	**0.001**			$X_{7,105}$ = 6.69	0.462	$X_{6,143}$ = 5.39	0.495

Table S4. Summary of pairwise contrasts comparing different sample preservation conditions against the control condition (i.e., snap freezing in liquid nitrogen, stored at -80ºC and measured three weeks later) for the antioxidant enzyme glutathione reductase (GR) in amphibian, insect, mammal, and bird samples. For each group, results are presented by tissue type, including the *t*-ratio and the corrected false-discovery-rate *P*-value for each comparison.

GR contrasts	Amphibian			Insect			Mammal			Bird		
	Tissue	t-ratio	P-value	Tissue	t-ratio	P-value	Tissue	t-ratio	P-value	Tissue	t-ratio	P-value
**-80ºC control vs -80ºC 8 months**	Liver	-2.82	**0.006**	Whole-body	-2.18	0.077	Liver	0.02	0.987	Liver	0.35	0.730
**-80ºC control vs 4ºC 2 hours**	Liver	-2.15	**0.033**	Whole-body	-1.51	0.192	Liver	0.02	0.981	Liver	0.01	0.989
**-80ºC control vs 4ºC 6 hours**	Liver	-0.13	0.893	Whole-body	-2.00	0.087	Liver	0.01	0.992	Liver	0.07	0.946
**-80ºC control vs 4ºC 24 hours**	Liver	-1.92	0.057	Whole-body	-2.63	0.074	Liver	0.01	0.992	Liver	0.02	0.980
**-80ºC control vs -20ºC 1 week**	Liver	-4.06	**<0.001**	Whole-body	1.04	0.354	Liver	0.02	0.984	Liver	-0.02	0.988
**-80ºC control vs -20ºC 2 months**	Liver	-3.93	**<0.001**	Whole-body	-2.19	0.077	Liver	0.02	0.983			
**-80ºC control vs -20ºC 8 months**	Liver	-4.34	**<0.001**	Whole-body	0.42	0.676	Liver	2.18	**0.031**	Liver	-0.01	0.990
**-80ºC control vs -80ºC 8 months**	Muscle	1.57	0.118				Muscle	1.04	0.301	Muscle	-2.46	**0.015**
**-80ºC control vs 4ºC 2 hours**	Muscle	0.11	0.909				Muscle	1.19	0.236	Muscle	-2.48	**0.015**
**-80ºC control vs 4ºC 6 hours**	Muscle	0.44	0.658				Muscle	0.70	0.484	Muscle	-2.53	**0.013**
**-80ºC control vs 4ºC 24 hours**	Muscle	0.96	0.336				Muscle	0.65	0.519	Muscle	-2.37	**0.019**
**-80ºC control vs -20ºC 1 week**	Muscle	0.48	0.631				Muscle	2.05	**0.043**	Muscle	-2.61	**0.010**
**-80ºC control vs -20ºC 2 months**	Muscle	-0.01	0.991				Muscle	1.03	0.305			
**-80ºC control vs -20ºC 8 months**	Muscle	0.59	0.555				Muscle	3.20	**0.002**	Muscle	-2.51	**0.013**

Table S5. Summary of the linear models (mixed for all species except for beetle samples) testing for the effects of storage conditions, tissue type, and their interaction on the activity of the enzyme superoxide dismutase (SOD) in amphibian, insect, mammal, and bird samples.

Superoxide dismutase	Amphibian		Insect		Mammal		Bird	
	Chi-sq	P-value	Chi-sq	P-value	Chi-sq	P-value	Chi-sq	P-value
**Storage conditions**	$X_{7,153}$ = 121.53	**<0.001**	$X_{7,73}$ = 33.75	**<0.001**	$X_{7,152}$ = 15.62	0.029	$X_{6,153}$ = 8.13	0.228
**Tissue**	$X_{1,159}$ = 183.21	**<0.001**			$X_{1,159}$ = 316.43	**<0.001**	$X_{1,159}$ = 6.61	0.010
**Storage conditions * Tissue**	$X_{7,153}$ = 116.03	**<0.001**			$X_{7,152}$ = 7.53	0.376	$X_{6,153}$ = 3.70	0.716

Table S6. Summary of pairwise contrasts comparing different sample preservation conditions against the control condition (i.e., snap freezing in liquid nitrogen, stored at -80ºC and measured three weeks later) for the antioxidant enzyme superoxide dismutase (SOD) in amphibian, insect, mammal, and bird samples. For each group, results are presented by tissue type, including the *t*-ratio and the corrected false-discovery-rate *P*-value for each comparison.

SOD contrasts	Amphibian			Insect			Mammal			Bird		
	Tissue	t-ratio	P-value	Tissue	t-ratio	P-value	Tissue	t-ratio	P-value	Tissue	t-ratio	P-value
**-80ºC control vs -80ºC 8 months**	Liver	-0.95	0.342	Whole-body	9.70	**<0.001**	Liver	1.32	0.189	Liver	-0.55	0.581
**-80ºC control vs 4ºC 2 hours**	Liver	-0.04	0.965	Whole-body	8.91	**<0.001**	Liver	0.38	0.708	Liver	-0.74	0.461
**-80ºC control vs 4ºC 6 hours**	Liver	0.56	0.573	Whole-body	8.33	**<0.001**	Liver	1.34	0.183	Liver	-0.43	0.668
**-80ºC control vs 4ºC 24 hours**	Liver	0.07	0.945	Whole-body	6.18	**<0.001**	Liver	1.28	0.203	Liver	-0.44	0.659
**-80ºC control vs -20ºC 1 week**	Liver	-0.62	0.534	Whole-body	1.41	0.189	Liver	1.03	0.306	Liver	-0.97	0.332
**-80ºC control vs -20ºC 2 months**	Liver	-0.41	0.681	Whole-body	8.66	**<0.001**	Liver	0.57	0.573			
**-80ºC control vs -20ºC 8 months**	Liver	1.84	0.068	Whole-body	0.66	0.514	Liver	1.45	0.150	Liver	-0.73	0.469
**-80ºC control vs -80ºC 8 months**	Muscle	-3.30	**0.001**				Muscle	-0.22	0.823	Muscle	-2.71	**0.008**
**-80ºC control vs 4ºC 2 hours**	Muscle	-3.09	**0.002**				Muscle	0.08	0.938	Muscle	-2.75	**0.007**
**-80ºC control vs 4ºC 6 hours**	Muscle	-3.53	**0.001**				Muscle	0.79	0.429	Muscle	-2.58	**0.011**
**-80ºC control vs 4ºC 24 hours**	Muscle	-2.35	**0.020**				Muscle	-0.22	0.825	Muscle	-2.43	**0.017**
**-80ºC control vs -20ºC 1 week**	Muscle	-3.10	**0.002**				Muscle	1.87	0.063	Muscle	-2.12	**0.036**
**-80ºC control vs -20ºC 2 months**	Muscle	8.35	**<0.001**				Muscle	-0.38	0.702			
**-80ºC control vs -20ºC 8 months**	Muscle	0.79	0.431				Muscle	2.82	**0.005**	Muscle	-1.85	0.066

Table S7. Summary of the linear models (mixed for all species except for beetle samples) testing for the effects of storage conditions, tissue type, and their interaction on the activity of the enzyme glutathione peroxidase (GPX) in amphibian, insect, mammal, and bird samples.

Glutathione peroxidase	Amphibian		Insect		Mammal		Bird	
	Chi-sq	P-value	Chi-sq	P-value	Chi-sq	P-value	Chi-sq	P-value
**Storage conditions**	$X_{7,152}$ = 6.88	0.441	$X_{7,55}$ = 3.46	**0.004**	$X_{7,153}$ = 16.19	**0.023**	$X_{6,153}$ = 14.82	**0.021**
**Tissue**	$X_{1,158}$ = 15.79	**<0.001**			$X_{1,159}$ = 1306.62	**<0.001**	$X_{1,159}$ = 971.54	**<0.001**
**Storage conditions * Tissue**	$X_{7,152}$ = 10.22	0.176			$X_{7,152}$ = 20.66	**0.005**	$X_{6,153}$ = 4.24	0.644

Table S8. Summary of pairwise contrasts comparing different sample preservation conditions against the control condition (i.e., snap freezing in liquid nitrogen, stored at -80ºC and measured three weeks later) for the antioxidant enzyme glutathione peroxidase (GPX) in amphibian, insect, mammal, and bird samples. For each group, results are presented by tissue type, including the *t*-ratio and the corrected false-discovery-rate *P*-value for each comparison.

GPX contrasts	Amphibian			Insect			Mammal			Bird		
	Tissue	t-ratio	P-value	Tissue	t-ratio	P-value	Tissue	t-ratio	P-value	Tissue	t-ratio	P-value
**-80ºC control vs -80ºC 8 months**	Liver	0.28	0.780	Whole-body	-1.25	0.380	Liver	-1.36	0.177	Liver	-2.31	**0.022**
**-80ºC control vs 4ºC 2 hours**	Liver	0.44	0.663	Whole-body	-0.81	0.594	Liver	-0.05	0.957	Liver	-1.21	0.228
**-80ºC control vs 4ºC 6 hours**	Liver	-0.39	0.696	Whole-body	-2.51	0.105	Liver	-0.55	0.585	Liver	-2.72	**0.007**
**-80ºC control vs 4ºC 24 hours**	Liver	-1.08	0.281	Whole-body	-0.48	0.632	Liver	0.60	0.551	Liver	-2.76	**0.007**
**-80ºC control vs -20ºC 1 week**	Liver	1.45	0.148	Whole-body	1.61	0.264	Liver	-0.12	0.905	Liver	-0.66	0.513
**-80ºC control vs -20ºC 2 months**	Liver	0.19	0.852	Whole-body	-1.89	0.224	Liver	-1.30	0.195			
**-80ºC control vs -20ºC 8 months**	Liver	-0.69	0.492	Whole-body	0.62	0.626	Liver	-3.67	**<0.001**	Liver	-0.85	0.394
**-80ºC control vs -80ºC 8 months**	Muscle	-0.10	0.919				Muscle	-0.31	0.757	Muscle	-1.62	0.108
**-80ºC control vs 4ºC 2 hours**	Muscle	0.08	0.933				Muscle	-0.22	0.828	Muscle	-1.61	0.110
**-80ºC control vs 4ºC 6 hours**	Muscle	2.00	**0.048**				Muscle	-0.53	0.597	Muscle	-1.61	0.110
**-80ºC control vs 4ºC 24 hours**	Muscle	-0.16	0.871				Muscle	-0.95	0.344	Muscle	-1.70	0.092
**-80ºC control vs -20ºC 1 week**	Muscle	-0.10	0.917				Muscle	0.09	0.928	Muscle	-1.03	0.304
**-80ºC control vs -20ºC 2 months**	Muscle	-0.69	0.493				Muscle	2.03	**0.044**			
**-80ºC control vs -20ºC 8 months**	Muscle	-0.17	0.869				Muscle	-0.50	0.620	Muscle	-1.93	0.055

Journal of Experimental Biology • Supplementary information

Table S9. Summary of the linear models (mixed for all species except for beetle samples) testing for the effects of storage conditions, tissue type, and their interaction on the concentration of malondialdehyde (MDA) in amphibian, insect, mammal, and bird samples.

Malondialdehyde	Amphibian		Insect		Mammal		Bird	
	Chi-sq	P-value	Chi-sq	P-value	Chi-sq	P-value	Chi-sq	P-value
**Storage conditions**	$X_{7,151} = 50.62$	**<0.001**	$X_{7,73} = 341.28$	**<0.001**	$X_{7,147} = 28.78$	**<0.001**	$X_{7,154} = 41.59$	**<0.001**
**Tissue**	$X_{1,157} = 24.94$	**<0.001**			$X_{1,143} = 1.59$	0.208	$X_{1,159} = 6.63$	**0.001**
**Storage conditions * Tissue**	$X_{7,151} = 42.92$	**<0.001**			$X_{7,147} = 52.98$	**<0.001**	$X_{6,152} = 30.55$	**<0.001**

Table S10. Summary of pairwise contrasts comparing different sample preservation conditions against the control condition (i.e., snap freezing in liquid nitrogen, stored at -80ºC and measured three weeks later) for the concentration of malondialdehyde (MDA) in amphibian, insect, mammal, and bird samples. For each group, results are presented by tissue type, including the *t*-ratio and the corrected false-discovery-rate *P*-value for each comparison.

MDA contrasts	Amphibian			Insect			Mammal			Bird		
	Tissue	t-ratio	P-value	Tissue	t-ratio	P-value	Tissue	t-ratio	P-value	Tissue	t-ratio	P-value
**-80ºC control vs -80ºC 8 months**	Liver	-1.46	0.147	Whole-body	-8.93	**<0.001**	Liver	-0.94	0.348	Liver	-0.46	0.645
**-80ºC control vs 4ºC 2 hours**	Liver	-1.33	0.186	Whole-body	-9.23	**<0.001**	Liver	-0.83	0.409	Liver	4.69	**<0.001**
**-80ºC control vs 4ºC 6 hours**	Liver	-0.24	0.815	Whole-body	-11.49	**<0.001**	Liver	-0.60	0.552	Liver	2.25	**0.026**
**-80ºC control vs 4ºC 24 hours**	Liver	0.33	0.741	Whole-body	-11.74	**<0.001**	Liver	1.25	0.214	Liver	-0.30	0.765
**-80ºC control vs -20ºC 1 week**	Liver	-1.01	0.313	Whole-body	-6.73	**<0.001**	Liver	0.06	0.950	Liver	-1.16	0.246
**-80ºC control vs -20ºC 2 months**	Liver	2.39	**0.018**	Whole-body	-9.61	**<0.001**	Liver	0.62	0.536			
**-80ºC control vs -20ºC 8 months**	Liver	5.27	**<0.001**	Whole-body	0.32	0.750	Liver	1.28	0.204	Liver	0.3	0.765
**-80ºC control vs -80ºC 8 months**	Muscle	-1.18	0.240				Muscle	2.66	**0.009**	Muscle	1.92	0.057
**-80ºC control vs 4ºC 2 hours**	Muscle	-0.85	0.397				Muscle	5.06	**<0.001**	Muscle	0.43	0.666
**-80ºC control vs 4ºC 6 hours**	Muscle	1.42	0.159				Muscle	2.77	**0.006**	Muscle	1.99	**0.048**
**-80ºC control vs 4ºC 24 hours**	Muscle	2.19	**0.030**				Muscle	6.31	**<0.001**	Muscle	-0.78	0.438
**-80ºC control vs -20ºC 1 week**	Muscle	-1.28	0.204				Muscle	1.18	0.240	Muscle	-0.01	0.993
**-80ºC control vs -20ºC 2 months**	Muscle	-0.85	0.395				Muscle	0.01	0.989			
**-80ºC control vs -20ºC 8 months**	Muscle	-0.11	0.910				Muscle	2.31	**0.023**	Muscle	-1.29	0.198

Table S11. Summary of the linear models (mixed for all species except for beetle samples) testing for the effects of storage conditions, tissue type, and their interaction on the activity of triglyceride concentration in amphibian, insect, mammal, and bird samples.

Triglyceride	Amphibian		Insect		Mammal		Bird	
	Chi-sq	P-value	Chi-sq	P-value	Chi-sq	P-value	Chi-sq	P-value
**Storage conditions**	$X_{7,153}$ = 17.91	**0.012**	$X_{7,73}$ = 28.56	<0.001	$X_{7,154}$ = 4.37	0.736	$X_{6,154}$ = 3.12	0.793
**Tissue**	$X_{1,158}$ = 165.43	**<0.001**			$X_{1,159}$ = 8.99	**0.003**	$X_{1,59}$ = 21.42	**<0.001**
**Storage conditions * Tissue**	$X_{7,153}$ = 14.55	**<0.004**			$X_{7,154}$ = 26.38	**<0.001**	$X_{6,153}$ = 10.33	0.111

Table S12. Summary of pairwise contrasts comparing different sample preservation conditions against the control condition (i.e., snap freezing in liquid nitrogen, stored at -80ºC and measured three weeks later) for triglyceride concentration in amphibian, insect, mammal, and bird samples. For each group, results are presented by tissue type, including the *t*-ratio and the corrected false-discovery-rate *P*-value for each comparison.

Triglyceride contrasts	Amphibian			Insect			Mammal			Bird		
	Tissue	t-ratio	P-value	Tissue	t-ratio	P-value	Tissue	t-ratio	P-value	Tissue	t-ratio	P-value
**-80ºC control vs -80ºC 8 months**	Liver	2.33	**0.022**	Whole-body	-7.72	**<0.001**	Liver	0.20	0.845	Liver	1.41	0.160
**-80ºC control vs 4ºC 2 hours**	Liver	2.48	**0.014**	Whole-body	-7.82	**<0.001**	Liver	0.49	0.625	Liver	1.86	0.065
**-80ºC control vs 4ºC 6 hours**	Liver	2.17	**0.031**	Whole-body	-8.16	**<0.001**	Liver	1.19	0.236	Liver	1.65	0.101
**-80ºC control vs 4ºC 24 hours**	Liver	2.31	**0.022**	Whole-body	-5.60	**<0.001**	Liver	2.62	**0.010**	Liver	2.09	0.039
**-80ºC control vs -20ºC 1 week**	Liver	2.34	**0.021**	Whole-body	-0.98	0.385	Liver	-0.03	0.973	Liver	2.73	**0.007**
**-80ºC control vs -20ºC 2 months**	Liver	3.13	**0.002**	Whole-body	-6.51	**<0.001**	Liver	1.14	0.257			
**-80ºC control vs -20ºC 8 months**	Liver	5.27	**<0.001**	Whole-body	0.73	0.470	Liver	2.16	**0.032**	Liver	2.51	**0.013**
**-80ºC control vs -80ºC 8 months**	Muscle	0.68	0.499				Muscle	1.33	0.186	Muscle	-1.05	0.297
**-80ºC control vs 4ºC 2 hours**	Muscle	0.64	0.524				Muscle	0.18	0.859	Muscle	-1.60	0.113
**-80ºC control vs 4ºC 6 hours**	Muscle	1.04	0.302				Muscle	-0.49	0.625	Muscle	-0.77	0.440
**-80ºC control vs 4ºC 24 hours**	Muscle	0.46	0.648				Muscle	-2.61	**0.010**	Muscle	-1.32	0.189
**-80ºC control vs -20ºC 1 week**	Muscle	0.75	0.455				Muscle	-0.59	0.553	Muscle	-0.82	0.411
**-80ºC control vs -20ºC 2 months**	Muscle	-0.48	0.632				Muscle	-0.54	0.593			
**-80ºC control vs -20ºC 8 months**	Muscle	0.68	0.496				Muscle	-0.32	0.751	Muscle	-0.99	0.326
